# Protective effects of sirtuin 3 in a murine model of sepsis-induced acute kidney injury

**DOI:** 10.1038/srep33201

**Published:** 2016-09-13

**Authors:** Wen-Yu Zhao, Lei Zhang, Ming-Xing Sui, You-Hua Zhu, Li Zeng

**Affiliations:** 1 Department of Organ Transplantation, Changhai Hospital, Second Military Medical University, Shanghai, PR China

## Abstract

Acute kidney injury (AKI) is a rapid loss of kidney function characterized by damage to renal tubular cells driven by mitochondrial dysregulation and oxidative stress. Here, we used a murine caecal ligation and puncture (CLP) model of sepsis-induced AKI to study the role of sirtuin 3 (SIRT3), a NAD^+^ dependent deacetylase critical for the maintenance of mitochondrial viability, in AKI-related renal tubular cell damage and explored the underlying mechanisms. CLP induced alterations in kidney function and morphology were associated with SIRT3 downregulation, and SIRT3 deletion exacerbated CLP-induced kidney dysfunction, renal tubular cell injury and apoptosis, mitochondrial alterations, and ROS production in a knockout mouse model. SIRT3 deletion increased the CLP-induced upregulation of the NLRP3 inflammasome and apoptosis-associated speck-like protein, resulting in the activation of oxidative stress, increased production of the proinflammatory cytokines interleukin (IL)-1β and IL-18, and the enhancement of apoptosis, and these effects were reversed by antioxidant NAC. Our results suggest that SIRT3 plays a protective role against mitochondrial damage in the kidney by attenuating ROS production, inhibiting the NRLP3 inflammasome, attenuating oxidative stress, and downregulating IL-1β and IL-18.

Acute kidney injury (AKI) is a rapid deterioration of kidney function that comprises ischemic, nephrotoxic, and septic components. AKI occurs in up to 7% of hospitalized patients and in 25% of patients in intensive care units, and is a major public health concern, with a high mortality rate that ranges from 50% to 80%^1^,^2^. AKI is characterized by damage to renal tubular cells, which are rich in mitochondria, and mitochondrial alterations are a hallmark of AKI^3^. Mitochondria are particularly susceptible to injury because of increased production of reactive oxygen species (ROS) and decreased antioxidant defences. The viability of mitochondria is largely maintained by Sirtuin 3 (SIRT3), a member of a conserved family of NAD^+^ dependent deacetylases that is synthesized as an inactive protein and is proteolytically processed to its active 28 KDa form during its translocation to the mitochondria^4^,^5^. SIRT3 overexpression in the kidneys reduces ROS and ameliorates mitochondrial dynamics^4^, suggesting that SIRT3 could be a master regulator of injury and repair in AKI.

Kidney injury involves morphological and functional changes in endothelial cells that trigger the infiltration of neutrophils, macrophages, natural killer cells and lymphocytes into the injured kidneys and the release of inflammatory mediators by tubular and endothelial cells^6^. Activation of the innate immune system in AKI involves the inflammasome, a multiprotein complex that activates the proinflammatory cytokines interleukin (IL)-1β and IL-18^7^,^8^. The nucleotide-binding domain (NOD)-like receptor protein 3 (NLRP3), which is the best characterized inflammasome, oligomerizes in response to stimulation, recruiting apoptosis-associated speck-like protein (ASC) to activate caspase-1^9^. Caspase-1 is a cysteine protease involved in the induction of apoptosis that plays a proinflammatory role by mediating the processing of IL-1β and IL-18 to their mature forms^10^.

Creatinine, a breakdown product of creatine phosphate that is removed from the blood by the kidneys, and blood urea nitrogen (BUN), a nitrogenous end product of protein and amino acid catabolism that is filtered by glomeruli, are the most commonly used markers of kidney function^11^. Elevated levels of creatinine and BUN are indicative of kidney disease or failure when correlated with glomerular filtration rates. Sepsis is a common cause of AKI, and the pathogenesis of sepsis-induced AKI involves inflammation, oxidative stress, and the responses of tubular epithelial cells. In the present study, the role of SIRT3 in mitochondrial damage associated with AKI was examined using a caecal ligation and puncture (CLP) model of sepsis-induced AKI in a SIRT3 knockout mouse model. Our results suggest that SIRT3 plays a protective role in the kidney mediated by the attenuation of ROS production and NLRP3 activity, suggesting potential therapeutic targets for the treatment of AKI.

## Results

### SIRT3 plays a role in CLP induced kidney damage

**Figure 1:**
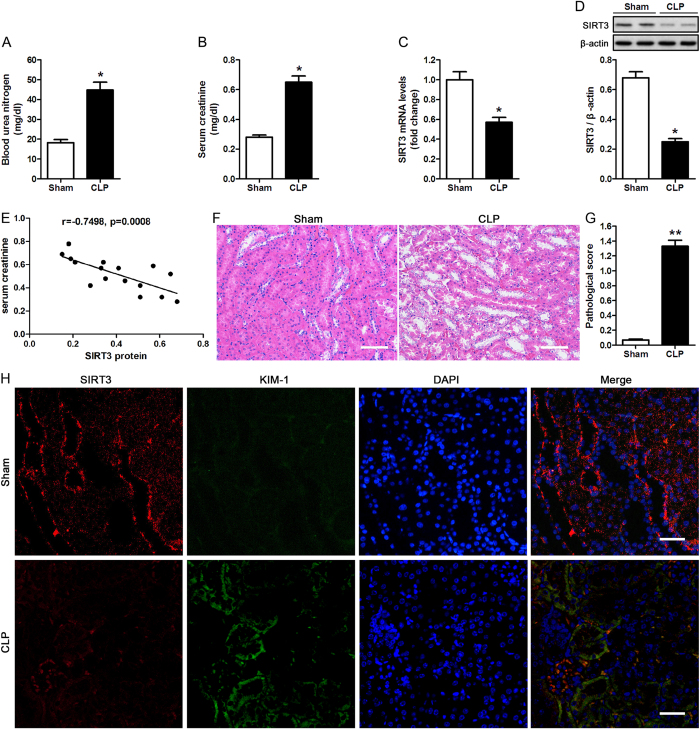
Caecal ligation and puncture (CLP) impairs kidney function and induces renal tubular damage. Blood samples and kidney tissues from male C57BL/6 mice were collected at 24 h after caecal ligation and puncture (CLP). Sham-operated animals served as negative controls (Sham). (**A**) Blood urea nitrogen (BUN) and (**B**) serum creatinine were analyzed using commercial kits. Whole kidney expression of SIRT3 mRNA (**C**) and protein (**D**) were analyzed by real-time PCR and western blotting, respectively. Data were normalized to GAPDH or β-actin. (**E**) Spearman’s correlation analysis was used to evaluate the relationship between the serum creatinine and SIRT3 protein level. (**F**) The collected kidneys were stained with haematoxylin and eosin (H&E). Scale bars: 50 μm. (**G**) Quantitative evaluation of morphological tubular damage. Data were expressed as the mean ± SEM (n = 8 mice/group). *p < 0.05, **p < 0.01 *versus* Sham. (**H**) Dual labeling of SIRT3 and KIM-1 in mice kidney. The nuclei were determined by DAPI labeling. Scale bars: 25 μm.

**Figure 2:**
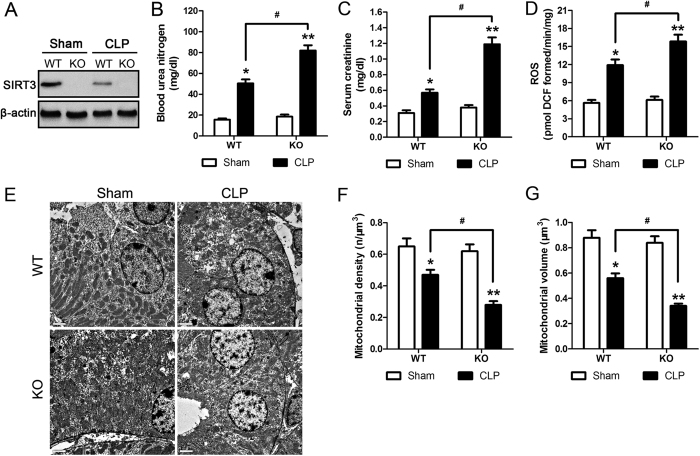
SIRT3 protects against CLP induced mitochondrial alterations and ROS production. Wild type (WT) and genetic deletion of SIRT3 (KO) mice received CLP surgery; sham-operated animals served as negative controls (Sham). Kidney tissues were collected at 24 h after CLP surgery. (**A**) SIRT3 protein of kidney tissues were analyzed by western blotting. (**B**) Blood urea nitrogen (BUN) and (**C**) serum creatinine were analyzed using commercial kits. (**D**) Levels of ROS in the kidney were determined using DCFH-DA. (**E**) Representative transmission electron micrographs of the ultrastructure of mouse proximal tubular cells obtained from resin-embedded kidney sections. Scale bars: 2 μm. Quantification of mitochondria per volume (**F**) and mean mitochondrial volume (**G**) by morphometric analysis in proximal tubular cells. All data were expressed as the mean ± SEM (n = 5–10 mice/group). *p < 0.05, **p < 0.01 *versus* Sham in WT or KO mice. ^#^p < 0.05.

The effect of CLP on kidney function and structure was investigated by real-time PCR and western blotting in blood samples and kidney tissues from male C57BL/6 mice subjected to CLP. BUN and serum creatinine levels were significantly higher in CLP than in Sham operated mice (Fig. 1A,B). CLP significantly downregulated SIRT3 at the mRNA and protein levels (Fig. 1C,D). Spearman analysis further revealed that SIRT3 protein level inversely correlated with serum creatinine (Fig. 1E), confirming the involvement of SIRT3 in AKI. Haematoxylin and eosin staining (H&E) of kidney tissue samples and quantification of tubular damage showed that CLP significantly induced vacuolar degeneration in the renal tubular epithelial cells and occasional neutrophil infiltration around glomeruli and in the interstitium (Fig. 1F,G). Double immunofluorescence staining with SIRT3 and kidney injury molecule 1 (KIM-1) showed that KIM-1 was upregulated concomitant with the downregulation of SIRT3 in response to CLP (Fig. 1H). The association between SIRT3 downregulation and CLP-induced renal functional and morphological injury suggested that SIRT3 plays a role in AKI. To further examine the role of SIRT3, kidney function and morphology were assessed in SIRT3 knockout mice (KO) in comparison to their wild-type counterparts (WT). SIRT3 downregulation in KO mice was confirmed by western blotting (Fig. 2A). The CLP-induced increase in BUN and serum creatinine levels was significantly higher in KO than in WT mice (Fig. 2B–C). These results indicated that SIRT3 may have a protective effect against sepsis-related alterations in kidney function and morphology and renal tubular cell viability.

### SIRT3 has a protective role against CLP induced mitochondrial alterations and ROS production

Based on the role of SIRT3 in the maintenance of mitochondrial integrity and the importance of mitochondrial integrity in AKI, we compared mitochondrial structure and function between WT and SIRT3 KO mice subjected to CLP. The CLP-induced increase in ROS production was significantly higher in KO than in WT mice (Fig. 2D). Figure 2E shows representative transmission electron micrographs of mouse proximal tubular cells from WT and KO mice subjected to Sham operation or CLP. The CLP-induced decrease in mitochondrial density and volume was significantly enhanced in mice with SIRT3 deletion compared with WT mice (Fig. 2F,G). These results indicated that SIRT3 deletion enhanced kidney injury-related ROS production and mitochondrial alterations, confirming the protective role of SIRT3 in the kidneys.

### Effect of SIRT3 deletion on CLP-induced NLRP3 inflammasome responses

**Figure 3:**
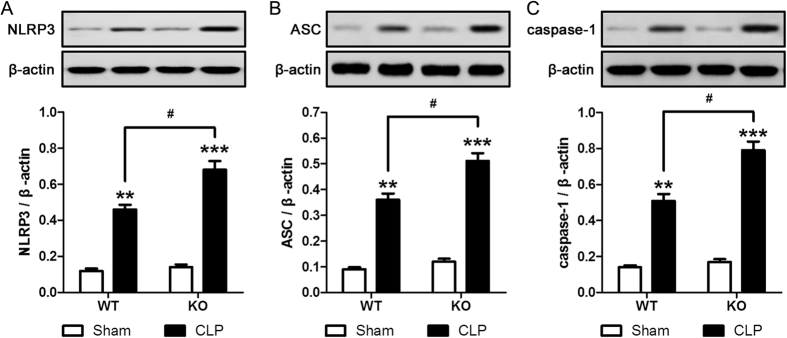
SIRT3 knockout exacerbates CLP induced NLRP3 inflammasome responses. Wild type (WT) and genetic deletion of SIRT3 (KO) mice received CLP surgery; sham-operated animals served as negative controls (Sham). Kidney tissues were collected at 24 h after CLP surgery. Western blot analysis of the protein levels of NLRP3 (**A**), ASC (**B**), and activated (cleaved) caspase-1 (**C**) in the whole kidney. The relative protein levels were determined after normalization to β-actin. All data were expressed as the mean ± SEM (n = 5–10 mice/group). **p < 0.01, ***p < 0.001 *versus* Sham in WT or KO mice. ^#^p < 0.05.

Given the role of the NLRP3 inflammasome in sepsis-induced AKI, we investigated whether SIRT3 knockout had an effect on AKI-related inflammatory responses by assessing NLRP3 levels and function in response to CLP. Our results showed that CLP induced NLRP3 upregulation and this effect was significantly greater in SIRT3 KO than in WT mice (Fig. 3A). Consistent with these results, CLP-induced upregulation of ASC and caspase-1 was significantly higher in KO mice than in WT mice (Fig. 3B,C), confirming that SIRT3 protects kidney tissues from sepsis-induced inflammatory injury.

### SIRT3 serves as an inflammatory regulator through the modulation of oxidation reaction

**Figure 4:**
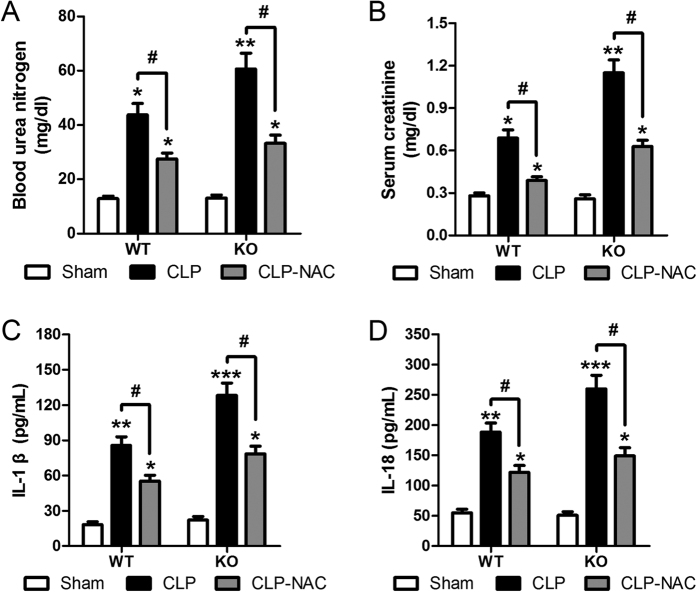
SIRT3 protects the kidneys against inflammation-related injury mediated by oxidative stress of proinflammatory cytokines. Wild type (WT) and SIRT3 knockout (KO) mice were injected intraperitoneally (i.p.) with either the anti-oxidant N-acetylcysteine (200 mg/kg, CLP-NAC) or isotonic saline in equivalent volumes at 12 h and 2 h before CLP. Sham-operated animals served as negative controls (Sham). Blood samples were collected at 24 h after CLP. (**A**) Blood urea nitrogen (BUN) and (**B**) serum creatinine were analyzed using commercial kits. Serum levels of IL-1β (**C**) and IL-18 (**D**) were measured by ELISA. All data were expressed as the mean ± SEM (n = 5–10 mice/group). *p < 0.05, **p < 0.01, ***p < 0.001 *versus* Sham in WT or KO mice. ^#^p < 0.05.

To further examine the relationship between ROS and inflammasome activation, we assessed the effect of the antioxidant NAC on CLP-induced AKI in WT and KO mice. Treatment of NAC partially restored BUN and serum creatinine levels increased by CLP in both WT and KO mice (Fig. 4A,B). Consistent with these results, the CLP-induced increase in IL-1β and IL-18 levels was significantly attenuated by pretreatment with NAC before CLP in both WT and KO mice (Fig. 4C,D). Taken together, these results indicated that SIRT3 protected against renal inflammation-related injury mediated by oxidation reaction.

**Figure 5:**
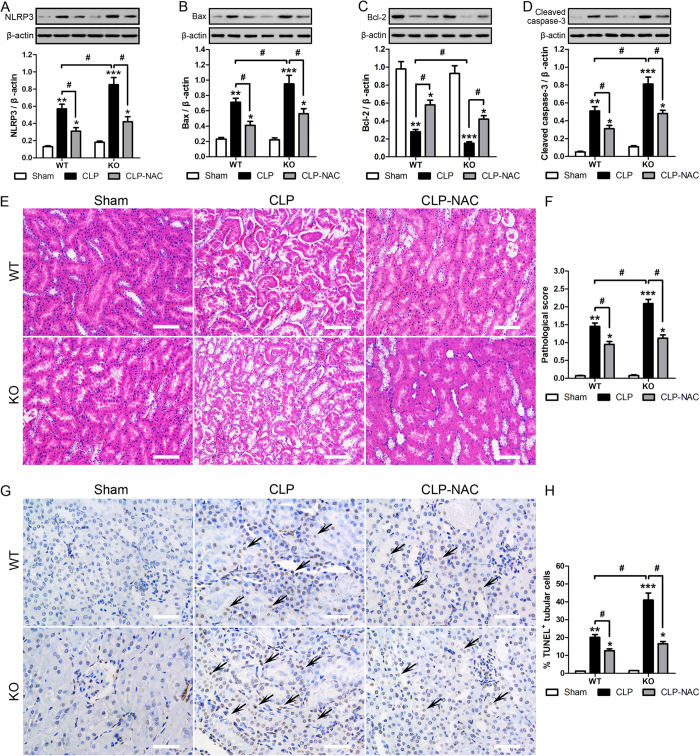
The protective role of SIRT3 is mediated by antioxidant activity. Wild type (WT) and/or SIRT3 knockout (KO) mice were treated (i.p.) with either 200 mg/kg NAC (CLP-NAC) or isotonic saline in equivalent volumes at 12 h and 2 h before CLP. Sham-operated animals served as negative controls (Sham). Kidney tissues were collected at 24 h after CLP surgery. Western blot analysis of the protein levels of NLRP3 (**A**), Bax (**B**), Bcl-2 (**C**) and cleaved caspase-3 (**D**) in the whole kidney. The relative protein levels were determined after normalization to β-actin. (**E**) The collected kidneys of mice were stained with H&E. Scale bars: 50 μm. (**F**) Quantitative evaluation of morphological tubular damage. (**G,H**) Apoptosis of renal tubular cells of mice was measured by TUNEL assay and quantified. Scale bars: 25 μm. All data were expressed as the mean ± SEM (n = 5–10 mice/group). *p < 0.05, **p < 0.01, ***p < 0.001 *versus* Sham in WT or KO mice. ^#^p < 0.05.

The role of SIRT3 in AKI was further examined by assessing inflammasome and apoptosis in relation to kidney tissue injury in our murine CLP model. Antioxidant NAC attenuated the CLP induced increase in inflammasome activation (Fig. 5A). Western blot analysis showed that NAC attenuated the CLP-induced upregulation of the pro-apoptotic proteins Bax and cleaved caspase 3 and the CLP-induced downregulation of the anti-apoptotic protein Bcl-2, restoring these proteins to similar levels in WT and KO mice (Fig. 5B–D). Figure 5E shows representative images of H&E stained kidney tissue samples in the different groups. The results showed that NAC inactivated the inflammasome and reduced renal injury, suggesting that mitochondrial damage and ROS production resulted in inflammasome activation. These results were verified by quantification of tubular damage and assessment of apoptosis by TUNEL staining, which showed that NAC significantly attenuated CLP-induced apoptosis of tubular cells in WT and SIRT3 KO mice (Fig. 5G,H). Taken together, these results indicated that the role of SIRT3 in regulating the inflammatory response may be mediated by the modulation of oxidation reaction.

### SIRT3 exerts a protective effect against oxidative stress *in vitro*

**Figure 6:**
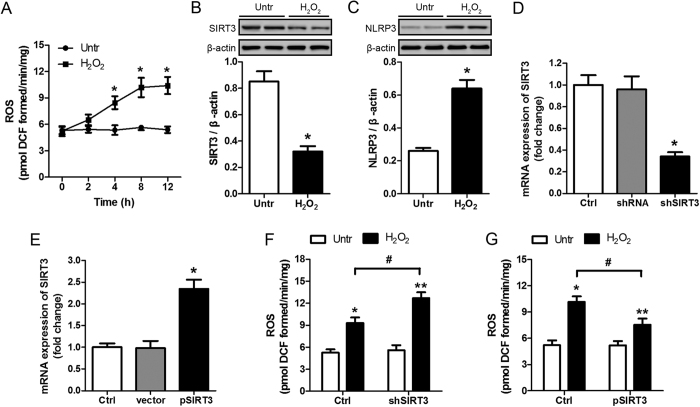
Effects of SIRT3 overexpression and silencing on oxidative stress induced damage. (**A**) HK-2 cells were stimulated by H_2_O_2_ (400 μM) at indicated time points. Intracellular production of ROS was determined using DCFH-DA. Data represent mean ± SEM of three separate experiments. *p < 0.05 *versus* untreated group (Untr). (**B**,**C**) HK-2 cells were treated with 400 μM H_2_O_2_ for 8 h. Western blot analysis of the protein levels of SIRT3 (**B**) and NLRP3 (**C**) in cells. Quantification of protein levels were performed by densitometry and normalized to the level of β-actin. Data represent mean ± SEM of three separate experiments. *p < 0.05 *versus* untreated group (Untr). (**D**,**E**) HK-2 cells were transduced with irrelevant shRNA (shRNA) or SIRT3 shRNA (shSIRT3), or with pBK-CMV vector (vector) or the vector expressing SIRT3 (pSIRT3). The non-transduced cells served as control (Ctrl). SIRT3 silencing (**D**) and overexpression (**E**) was confirmed on selected clones by real-time PCR of whole cells lysates. Data were normalized to GAPDH and depicted as fold change relative to the Ctrl group. Data represent mean ± SEM of three separate experiments. *p < 0.05 *versus* Ctrl group. (**F,G**) HK-2 cells transduced with shSIRT3 (**F**) or pSIRT3 (**G**) were treated with 400 μM H_2_O_2_ for 8 h. Intracellular production of ROS was determined using DCFH-DA. Data represent mean ± SEM of three separate experiments. *p < 0.05, **p < 0.01 *versus* Untr of each group. ^#^p < 0.05.

**Figure 7:**
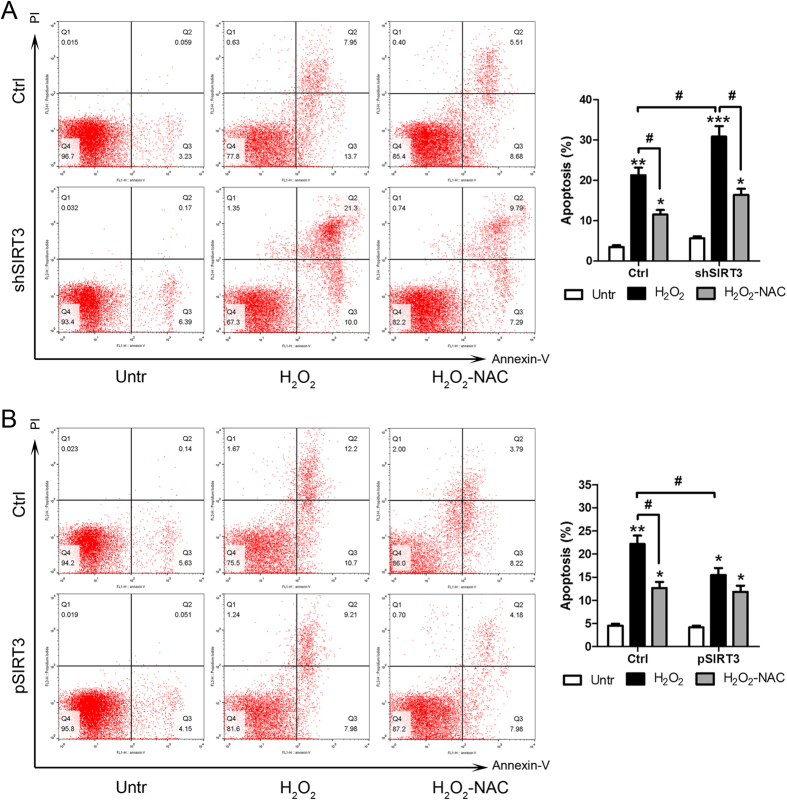
Effects of SIRT3 overexpression and silencing on apoptosis. After the HK-2 cells were transfected with shSIRT3 (**A**) or pSIRT3 (**B**), cells were pre-treated with NAC (10 mM, H_2_O_2_-NAC), or with vehicle control, for 2 h prior to the treatments of 400 μM H_2_O_2_ for 8 h. Cells were stained with Annexin V and propiumiodine and were subject to FACS. Bar graph summarizing the FACS results showing the population of Annexin V positive apoptotic cells. Data represent mean ± SEM of three separate experiments. *p < 0.05, **p < 0.01, ***p < 0.001 *versus* Untr of each group. ^#^p < 0.05.

To further determine the role of SIRT3 in AKI, the effects of SIRT3 overexpression and silencing in HK-2 cells were examined. Treatment of HK-2 cells with H_2_O_2_ significantly increased the production of ROS (Fig. 6A). H_2_O_2_ treatment significantly downregulated SIRT3 and upregulated NLRP3 (Fig. 6B,C). Figures 6D,E show the effective knockdown and overexpression of SIRT3 in HK-2 cells, as determined by RT-PCR. SIRT3 silencing enhanced ROS production in response to H_2_O_2_ treatment, whereas SIRT3 overexpression had the opposite effect (Fig. 6F,G). Analysis of apoptosis by flow cytometry showed that SIRT3 silencing significantly enhanced H_2_O_2_ induced apoptosis, and co-treatment with NAC partially restored this effect (Fig. 7A). Conversely, SIRT3 overexpression significantly inhibited H_2_O_2_ induced apoptosis in HK-2 cells (Fig. 7B). Taken together, these results confirm that SIRT3 protects against sepsis-induced AKI via the ROS/caspase pathway.

## Discussion

Reversible acetylation of the ε-amino group of lysine residues plays a critical role in the regulation of protein function and is catalyzed by the opposing activities of acetyltransferases and deacetylases^12^. The sirtuin family of deacetylases comprises seven members, of which the mitochondrial SIRTs play a role in metabolic homeostasis, and SIRT3 in particular regulates many mitochondrial processes and promotes cell survival by restricting ROS production and deacetylating cyclophilin D, a peptidyl-prolyl isomerase that activates the mitochondrial permeability transition pore^13^,^14^,^15^,^16^,^17^. Despite the crucial role of mitochondrial protein acetylation and extensive research into the roles of sirtuins, many aspects of sirtuin function and the regulation of sirtuin activity in mitochondria are not completely understood. Here, we showed that SIRT3 protects against sepsis-induced kidney alterations by attenuating inflammatory responses, modulating tubular cell apoptosis, and decreasing ROS-related mitochondrial damage.

In the present study, SIRT3 deletion exacerbated the CLP-induced increase in BUN and creatinine, renal tubular cell injury, and apoptosis, suggesting a protective role for SIRT3 in the kidneys. These results were consistent with those reported by Morigi *et al*., who showed that in mice with cisplatin-induced AKI, tubular cell mitochondrial abnormalities are associated with decreased levels of renal SIRT3, and restoration of SIRT3 expression and activity improves renal function^4^. The increase in ROS production and mitochondrial damage induced by SIRT3 deletion in our model suggested that SIRT3 protects the kidney from oxidant-related tissue injury and ROS-induced mitochondrial damage^18^. This is consistent with the role of SIRT3 in modulating ROS production in mitochondria by deacetylating mitochondrial complex I and II and ROS clearance by modulating the acetylation of superoxide dismutase (SOD)2, which scavenges ROS by catalyzing the conversion of superoxide to hydrogen peroxide^19^,^20^. SIRT3 was recently shown to attenuate ROS production induced by doxorubicin by upregulating SOD2, resulting in improved mitochondrial bioenergetics and suggesting a potential strategy for the treatment of doxorubicin-related cardiac dysfunction^21^. In the cisplatin-induced AKI model, treatment with an antioxidant restored SIRT3 expression and improved renal function^4^, supporting that the protective role of SIRT3 against kidney damage is mediated by its effect on oxidative stress-related mitochondrial damage.

ROS activate the NLRP3 inflammasome, and mitochondrial damage is associated with NLRP3 inflammasome activation, although the underlying mechanisms are not clear^22^. Our results suggested the involvement of the NLRP3 inflammasome in the protective effect of SIRT3, as indicated by increased NLRP3-mediated inflammatory responses in SIRT3 knockout mice and their reversal by antioxidant NAC. Sirtuin activation promotes anti-inflammatory responses, and decreased activity of sirtuins has been implicated in inflammation. SIRT2 inactivation results in the accumulation of acetylated α-tubulin, which associates with NLRP3, leading to its localization to the mitochondria, where it is activated in a ROS dependent manner^23^. SIRT1 has anti-inflammatory effects mediated by the deacetylation and inactivation of the p65 subunit of the proinflammatory transcription factor NF-κB and by decreasing ROS production through the activation of autophagy^24^,^25^. However, little information is available on the relation of SIRT3 with NLRP3. Furthermore, the localization of NLRP3 to mitochondria remains controversial^22^,^26^,^27^; therefore, a direct effect of SIRT3 on the NLRP3 inflammasome cannot be ruled out.

Crosstalk between sirtuins and the inflammasome has been studied mainly for its role in aging, and SIRT3 maintains the normal function of mitochondria by ensuring the scavenging of ROS to prevent cell senescence and apoptosis associated with increased ROS concentration^28^,^29^. However, the role of SIRT3 in apoptosis is not clear, as it has been shown to have pro-apoptotic and anti-apoptotic activity depending on cell type. Our results suggest that the anti-apoptotic effect of SIRT3 is mediated by its inhibition of ROS production leading to the inactivation of the NLRP3 inflammasome; however, a direct effect on mitochondrial apoptosis in renal tubular cells cannot be ruled out. SIRT3 overexpression was shown to block the translocation of Bax into mitochondria in cardiomyocytes^5^. SIRT3 overexpression rescued SIRT1-deficient HeLa cells from genotoxic stress-induced apoptosis, suggesting that its protective effect is mediated by the deacetylation of Ku70, which forms a complex with Bax when deacetylated, preventing its translocation to mitochondria and thereby blocking apoptosis^5^. In bladder carcinoma cells, SIRT3 was shown to deacetylate p53, which induces apoptosis when acetylated^30^. SIRT3 therefore induces survival and protects cells against damage by preserving the integrity of mitochondria or increasing their resistance to stress, and its overexpression suppresses apoptosis, supporting its role as an oncogene^31^. In the kidneys, the role of SIRT1 in protecting renal tubular cells against oxidative-stress-induced apoptosis has been demonstrated extensively^32^. However, little information is available on the role of SIRT3, although it was shown to exert antioxidant and anti-inflammatory effects by promoting mitochondrial antioxidant responses in proximal tubular cells^33^.

Extensive research on sirtuins supports their role as antioxidants that limit the activation of NLRP3, conferring protection against injury, as determined in the present study. Our results suggest a mechanism of action by which SIRT3 protects against tissue damage by attenuating ROS production, which reduces NLRP3 activity, resulting in the inhibition of oxidative stress and apoptosis and the downregulation of proinflammatory cytokines. However, further research is necessary to determine whether SIRT3 could have a direct effect on the NLRP3 inflammasome or apoptosis. Identification of the deacetylation target of SIRT3 specific to its renoprotective role may help elucidate its mechanism of action. For example, SIRT3 was shown to protect mitochondria against oxidative damage by deacetylating FOXO3, which modulates the expression of many genes, such as the antioxidant enzymes manganese superoxide dismutase and catalase^34^. In proximal tubular kidney cells, SIRT3 overexpression attenuated palmitate induced ROS accumulation and expression of the inflammatory cytokine monocyte chemoattractant protein-1, whereas these effects were enhanced by expression of a dominant negative form of SIRT3 or siRNA mediated SIRT3 knockdown^33^. Nevertheless, our results reveal a potential underlying mechanism for the protective effect of SIRT3 and highlight the therapeutic potential of SIRT3 and its effectors for the treatment of AKI.

## Methods

### Animal model of acute kidney injury

C57BL/6 mice (male, 8–10 weeks old) were purchased from Shanghai Laboratory Animal Center (SLAC, Shanghai, China); 10 week-old male 129sv-SIRT3 knockout (KO) and wild-type (WT) littermate mice were purchased from The Jackson Laboratory (Bar Harbor, ME, USA), and created by Lombard *et al*.^29^. All experiments were performed in accordance with the Chinese legislation on the use and care of laboratory animals and were approved by the Animal Care and Use Committee of Changhai hospital. CLP was performed as described previously^35^. Briefly, mice were anesthetised with isoflurane and a midline incision (2 cm) was made below the diaphragm to expose the caecum. The caecum was ligated at the colon juncture with a 5–0 silk ligature suture, punctured twice with a 21-gauge needle, laced back in the abdomen, and the incision was closed in two layers with a 5–0 silk ligature suture. Sham operation was performed in the same way as CLP, but without ligation and puncture of the caecum. Mice were fluid-resuscitated with 0.8 ml normal sterile saline injected subcutaneously. At 24 h after CLP surgery, blood was isolated by intracardiac puncture. Whole blood was stored overnight at 4 °C and the serum was isolated by centrifugation at 4000 × g for 10 min. Serum samples were frozen at −80 °C for subsequent use. For tissue analyses, kidneys were harvested, one third was fixed in 4% paraformaldehyde and processed for H&E staining and TUNEL staining, one third was fixed in glutaraldehyde for transmission electron microscopy analysis, and the other one third was snap-frozen in liquid nitrogen and stored at −80 °C for subsequent biochemical analysis.

### Experimental design

C57BL/6 mice were assigned to sham and CLP groups, n = 8 each. Wild type (WT) and genetic deletion of SIRT3 (KO) mice were randomly assigned to three groups as follows: Sham group (n = 5 for each WT and KO): mice receiving sham operation; CLP group (n = 20 for each WT and KO): mice receiving CLP operation with or without isotonic saline treatment; CLP-NAC (n = 10 for each WT and KO): mice subjected to CLP were pretreated with the anti-oxidant N-acetylcysteine (NAC, 200 mg/kg) administered intraperitoneally (i.p.) at 12 h and 2 h before CLP.

### Measurement of renal function

Renal function was evaluated by measuring BUN and serum creatinine levels using specific commercially available kits: BUN Assay Kit (Jiancheng Biotech, Nanjing, P. R. China) and Creatinine Assay Kit (Biosino Bio-Technology).

### Histology and tubular injury score

Formalin-fixed and paraffin-embedded kidney tissues were cut into 3-μm sections, stained with H&E, and visualized under an optical microscope (Olympus Optical, Tokyo, Japan). Tubular injury was scored from 0 to 3 as follows: 0 = normal histology; 1 = tubular cell swelling, brush border loss, nuclear condensation, with up to 1/3 of tubular profile showing nuclear loss; 2 = as for score 1, but greater than 1/3 and less than 2/3 of tubular profile shows nuclear loss; and 3 = greater than 2/3 of tubular profile shows nuclear loss.

### Cell culture and treatment

Human renal proximal tubular epithelial HK-2 cells were cultured in Dulbecco’s modified Eagle’s medium containing 10% foetal bovine serum and antibiotics. Cells were cultured to 80–90% confluency and then treated treated for 2–12 h with 400 μM H_2_O_2_ for oxidative stress. Cells were pre-treated with 10 mM NAC for 2 h prior to the treatments. DMSO was used as a vehicle control.

### SIRT3 silencing and overexpression

For SIRT3 silencing, cells were infected with Mission^TM^ TRC shRNA lentiviral transduction particles expressing short hairpin RNA targeting SIRT3 (shSIRT3) and lentiviral negative control particles (shRNA) purchased from Sigma–Aldrich Chemie GmbH. For SIRT3 overexpression, cells were transfected with expressing SIRT3 plasmid DNA using Lipofectamine 2000 (Invitrogen) according to the manufacturer’s instructions. The plasmid used was pBK-CMV expression vector (Stratagene, La Jolla, CA).

### Quantitative real-time PCR

Total RNA was extracted from kidney tissues and HK-2 cells using the TRIzol reagent (Invitrogen) according to the manufacturer’s instructions. First-strand cDNA was generated using a SuperScript VILO cDNA Synthesis Kit (Life Technologies) according to the manufacturer’s instructions. Quantitative real-time PCR was performed using SYBR Green PCR mix on an ABI Prism 7900HT (Applied Biosystems, Foster City, California, USA). Sequences of primers used for amplifications were as follows: mouse SIRT3, forward: 5′-TCA CAA CCC CAA GCC CTT TT-3′, reverse: 5′-GTG GGC TTC AAC CAG CTT TG-3′; mouse GAPDH, forward: 5′-TGA AGG GTG GAG CCA AAA GG-3′, reverse: 5′-GAT GGC ATG GAC TGT GGT CA-3′. Human SIRT3, forward: 5′-TCA CTA CTT TCT CCG GCT GC-3′, reverse: 5′-CAA TGT CGG GCT TCA CAA CG-3′; human GAPDH, forward: 5′-TGT TCG TCA TGG GTG TGA AC-3′, reverse: 5′-GCC AGT AGA GGC AGG GAT GA-3′. PCR conditions were 95 °C for 10 min, then 40 amplification cycles of 95 °C for 15 s and 60 °C for 1 min. The mRNA expression level was normalized by GAPDH. The relative mRNA expression was calculated using the 2^−△△CT^ method and presented as fold changes relative to control group.

### Western blotting

Kidney tissues or HK-2 cells were directly lysed with RIPA containing protease and phosphatase inhibitors (Roche, Basel, Switzerland). Total proteins were separated by 10% SDS-PAGE after denaturation and transferred onto nitrocellulose membranes. After blocking, the membranes were incubated with rabbit anti-mouse monoclonal antibodies against SIRT3, NLRP3, ASC, caspase-1, Bax, Bcl-2, caspase-3 or β-actin (1:400, Cell Signaling, Denvers, USA) overnight at 4 °C with shaking, and then incubated with the respective peroxidase-conjugated goat anti-rabbit IgG (1:2000, Santa Cruz, CA, USA) for 1 h at room temperature. The membranes were washed three times, and then detected using an enhanced chemiluminescence reagent kit (Amersham Life Science, Cleveland, USA). The gray values were determined by a gel image analysis system (Bio-Rad, Hercules, USA) and normalized to β-actin.

### Immunofluorescence

For immunofluorescence (IF), 2 μm acetone-fixed cryostat sections and 4 μm paraffin sections were cut from the snap-frozen human kidney specimens and methyl Carnoy’s solution-fixed rat kidneys, respectively. The sections were incubated with the following primary antibodies: goat polyclonal to SIRT3 (1:100, Santa Cruz) and rabbit polyclonal to KIM-1 (1:100, abcam). To detect the primary antibodies, the sections were incubated with rhodamine-conjugated anti-goat IgG (1:100, abcam) for SIRT3, fluorescein isothiocyanate (FITC)-conjugated anti-rabbit IgG (1:100, abcam) for KIM-1. The nuclei were stained with 4′,6′-diamidino-2-phenylindole hydrochloride (DAPI). Sections were visualized using a Laser-Scanning Confocal Microscope (Olympus FluoView™ FV1000, Tokyo, Japan).

### Terminal dUTP nick-end labelling staining

Paraffin-embedded sections were stained using the TUNEL technique using an *in situ* apoptosis detection kit (Promega) according to the manufacturer’s protocol. TUNEL-positive cells were counted in 12 randomly selected fields from each slide at a magnification of ×400, and the percentage of TUNEL positive cells was calculated from six kidney sections from different mice.

### Estimation of mitochondrial numerical density and mean mitochondrial volume

Glutaraldehyde-fixed kidney tissue sections were washed, postfixed in 1% OsO_4_, dehydrated through a graded alcohol series, and embedded in Epon resin. Sections were stained with uranyl acetate and examined using a Phillips Morgagni transmission electron microscope. The density of mitochondria (N_v_, n/μm^3^) was calculated using an orthogonal grid digitally superimposed onto digitized electron microscope images of proximal tubules at ×7,100 magnification as previously described^36^. Mitochondrial volume was calculated as the ratio of mitochondrial volume density to numerical density as described previously^4^.

### Assay of ROS level

ROS was measured as described previously, based on the oxidation of 2′7′- dichlorodihydrofluorescein diacetate (DCFH-DA) to 2′7′-dichloro-fluorescein (DCF) as described previously^37^. Briefly, tissue homogenates and cells were diluted 1:20 in Locke’s buffer (154 mM NaCl, 5.6 mM KCl, 3.6 mM NaHCO_3_, 2.0 mM CaCl_2_, 10 mM d-glucose, and 5 mM HEPES, pH 7.4) to obtain a concentration of 5 mg tissue/ml. The reaction mixture containing the homogenate and DCFH-DA (5 mM) was incubated for 15 min at room temperature for 30 min, and the conversion of DCFH-DA to the fluorescent product DCF was measured using a spectrofluorimeter with excitation at 484 nm and emission at 530 nm. ROS formation was quantified using a DCF-standard curve and data were expressed as pmol DCF/min/mg protein.

### Enzyme-linked immunosorbent assay

Serum levels of IL-1β and IL-18 were measured by enzyme-linked immunosorbent assay (ELISA) using specific kits (Biosource International Inc, Camarillo, CA) according to the manufacturer’s instructions.

### Apoptosis assay

Apoptosis was assessed by an Annexin V-FITC/PI Apoptosis Detection Kit (BD Pharmingen, CA, USA) according to the manufacturer’s instructions. Cells were cultured and treated as indicated. Cells were resuspended in binding buffer and incubated with Annexin V-FITC for 30 min at room temperature in the dark followed by PI staining. Cells were analyzed in a flow cytometer (FACScan, Becton Dickinson, USA) and analyzed using Cell Quest Software (Beckton Dickinson).

### Statistical analysis

Data were presented as the mean ± standard error. Results were analysed using Graphpad Prism 6 software (GraphPad Software Inc., La Jolla, USA). Differences between groups were evaluated using the Student’s t-test, and the significance among groups was determined using one-way ANOVA followed by Tukey’s post-hoc test. Correlation analysis between SIRT3 protein level and serum creatinine level was performed using two-tailed Spearman’s correlation coefficient (r). Statistical significance was set at P < 0.05.

## Additional Information

**How to cite this article**: Zhao, W.-Y. *et al*. Protective effects of sirtuin 3 in a murine model of sepsis-induced acute kidney injury. *Sci. Rep.* **6**, 33201; doi: 10.1038/srep33201 (2016).
